# Social Frailty in Late Adulthood: Social Cognitive and Psychological Well-Being Correlates

**DOI:** 10.1093/geronb/gbac157

**Published:** 2022-10-03

**Authors:** Julie D Henry, Sarah P Coundouris, Jessica Mead, Brielle Thompson, Ruth E Hubbard, Sarah A Grainger

**Affiliations:** School of Psychology, The University of Queensland, Brisbane, Queensland, Australia; School of Psychology, The University of Queensland, Brisbane, Queensland, Australia; School of Psychology, The University of Queensland, Brisbane, Queensland, Australia; School of Psychology, The University of Queensland, Brisbane, Queensland, Australia; Centre for Health Services Research, Faculty of Medicine, The University of Queensland, Brisbane, Queensland, Australia; School of Psychology, The University of Queensland, Brisbane, Queensland, Australia

**Keywords:** Frailty, Social cognition, Successful aging, Well-being

## Abstract

**Objectives:**

Social frailty poses a major threat to successful aging, but its social cognitive and psychological well-being correlates remain poorly understood. This cross-sectional study provides initial insights into whether social cognitive difficulties in older age are associated with social frailty, as well as how social frailty is linked to psychological characteristics known to be important for health and well-being.

**Method:**

Ninety community-dwelling older adults completed measures of social frailty and social cognition (social perception, theory of mind, affective empathy, and informant-rated social behavior) as well as measures of psychological function known to be important for health and well-being, both positively (resilience and life satisfaction) and negatively (demoralization, social anxiety, and apathy). Measures of cognitive frailty, physical frailty, and depression were also administered to test the specificity of any observed relationships with social frailty.

**Results:**

Both affective empathy and social behavior were predictive of increased social frailty, but social behavior emerged as the only unique predictor after controlling for covariates. Social frailty also predicted unique variance in all five measures of psychological well-being, and for three of these measures (demoralization, resilience, and life satisfaction), the effects remained significant even after adjusting for covariates.

**Discussion:**

Findings are discussed in relation to models of socioemotional aging and frailty. Potential mechanisms linking social behavior to social capital in older age are identified, as well as how loss of social resources might both directly and indirectly impact well-being.

In late adulthood, it is common for certain social needs to increase, with greater practical support required to manage the demands of everyday life due to decreased physical and cognitive capacity, and greater emotional support required to cope with bereavement (Lee et al., 2020). Yet relative to their younger counterparts, older adults are at heightened risk of social isolation, have fewer social ties, are more likely to be living alone, and engage in fewer social activities. It is this disparity between older adults’ increased social dependence on others and their reduced access to social resources that makes them particularly vulnerable to social frailty.

Frailty is now recognized to be a multidimensional construct that includes three distinct but interrelated components: physical, cognitive, and social frailty. Although the social component is the least well understood of the three, prevalence estimates in community-dwelling older adults are consistently high, ranging from 7.7% to 20.5% for social frailty, and from 25.0% to 32.1% for social pre-frailty (an early stage of social frailty; Ma et al., 2018; Yamada & Arai, 2018). Social frailty has also been consistently linked to negative outcomes in late adulthood. Perhaps most striking are studies showing that social frailty is associated with increased disability and mortality, even after adjusting for other vulnerability-related covariates such as physical frailty, cognitive frailty, and depression (Makizako et al., 2015; Yamada & Arai, 2018).

According to Bunt et al.’s (2017) model, social frailty occurs when basic social needs are either inadequately fulfilled or under threat of being inadequately fulfilled. This means that while vulnerability characteristics such as social isolation (a lack of social interaction) or subjective loneliness (the feeling that an individual’s social network is smaller or of poorer quality than preferred) may be considered symptoms of social frailty, they do not completely capture its meaning. Rather, fulfillment of social needs is reliant on three distinct but interrelated components: access to social resources (such as living offspring, a spouse, friends, or neighbors), social behaviors (such as volunteering, employment, and community engagement), and general resources (such as financial situation, living environment, and education), with social behaviors and social resources considered the most important of the three (Bunt et al., 2017).

Although at present the mechanistic factors that drive resilience and risk for social frailty remain poorly understood, for all three aspects of frailty the most widely accepted view is that the development of this vulnerability status represents the accumulation of “debt,” whereby physical, cognitive, and/or social difficulties or losses accrue, thereby reducing an individual’s reserves and resilience in the affected domain. Viewed in this context, the loss of social resources and social behaviors central to Bunt et al.’s (2017) framework may at least partially reflect an accumulation of “debt” in the specific domain of social cognition. This is because social cognition refers to how we perceive, interpret, and process social information about ourselves and others, and is critical to successfully communicate, connect, and relate to others in all areas of life—and to therefore retain or build social capital.

In daily life, social cognitive failures typically present in one of four ways (Henry et al., 2016): as problems recognizing social and emotional cues (social perception), as a reduced ability to understand the mental states of others (theory of mind [ToM]), as a muted or excessively strong emotional response to others (affective empathy), or as abnormal or inappropriate behavior (social behavior). (While poor social behavior in the context of Bunt’s model simply refers to a lack of engagement in activities that are social in nature, in the context of social cognition, poor social behavior instead refers to social behavioral abnormalities, such as poor social tact, a lack of manners, interpersonal boundary infringements, reduced use of communicative gestures and unsolicited affiliative contact with strangers.) Although most evidence points to stasis or even age-related gains in affective empathy (Beadle et al., 2015; Grainger et al., 2022), for each of the three other social cognitive domains age-related decline has been the most consistent finding. Age-related losses have been identified in key aspects of social perception such as gaze-cued attention (McKay et al., 2022) and facial affect recognition (Hayes et al., 2020), as well as in ToM understanding (Henry et al., 2013). Older (relative to younger) adults are also more likely to exhibit social behaviors that are considered inappropriate by their peers (Henry et al., 2009) and to endorse extremist attitudes that are aversive to others (Ruffman et al., 2016).

Yet there is surprisingly limited evidence that speaks directly to the real-life consequences of social cognitive failures in older age. This is in contrast with the literature on clinical populations, in which social cognitive difficulties have been consistently linked to negative outcomes, including less supportive social networks, unemployment, and poorer mental health (Henry et al., 2016). However, this clinical literature provides important proof of concept, as it suggests that if older adults do experience social cognitive difficulties, there may be meaningful costs. The first aim of the present study was therefore to establish whether one of these potential costs might be increased social frailty, by providing the first test of whether specific aspects of older adults’ social cognitive function are related to their level of social frailty.

It is also important to understand the psychological correlates of social frailty, particularly those that have previously been linked to negative health and well-being outcomes in older age, as this has implications for how the negative effects of social frailty might be reduced or even prevented. Yet most studies to date that have investigated the psychological correlates of social frailty have focused on mental illness, and in particular, depression. While undoubtedly important, this affords only a quite restricted view of the potential impact of social frailty on psychological well-being. The second aim was therefore to broaden this focus, to establish how social frailty is related to a more diverse range of indicators or predictors of psychological well-being. Because in older cohorts, positive and negative affect are differentially related to loneliness, companionship, and satisfaction with social activities (Davidson et al., 2022), positive (resilience and life satisfaction) and negative (demoralization, social anxiety, and apathy) indicators and predictors of psychological well-being were considered separately.

In sum, using a cross-sectional design, regression analyses were used to test the preregistered predictions that (a) poorer social perception, affective empathy, ToM, and social behavior will be predictive of elevated levels of social frailty, and (b) higher levels of social frailty will be predictive of lower resilience and life satisfaction, and greater demoralization, social anxiety, and apathy. The study will also explore whether each of these predicted relationships remain significant after adjusting for physical, cognitive, and mental health covariates previously linked to social frailty (physical frailty, cognitive frailty, and depression).

## Method

### Participants

No prior study has examined associations between social frailty and social cognition, but Pek al.’s (2020) study revealed a large-sized association between the Social Frailty Scale and well-being as indexed by a measure of depression. Because moderate-sized associations between social frailty and wellbeing indicators might also have potentially important practical implications, an a priori power analysis using G*POWER software was conducted to establish the minimum sample size needed to achieve excellent power (1 – β > 95%, α = 0.05) to detect a moderate-sized effect (*f* = .15). This revealed that 89 participants were required.

Ninety participants (50% male) aged between 65 and 91 years of age (*M* = 73.78, *SD* = 5.92) were recruited from a diverse range of community settings. Participants were eligible if they were 65 years of age or older, fluent in English, and had no diagnosis of neurocognitive impairment, major psychiatric illness, or neurodevelopmental disorders. All participants scored above the cut-off on the Mini Addenbrooke’s Cognitive Examination—Revised (Hsieh et al., 2015; *M* = 28.16, *SD* = 1.77). Demographic information is provided in Supplementary Table 1.

### Materials and Procedure

The study was approved by The University of Queensland Ethics Committee (HE000164), and the protocol was preregistered on the Open Science Framework (https://osf.io/27eky/?view_only=681b0813c2be4031ad3c33e085fa6e27).

Participants were tested individually face-to-face for approximately 60–90 min, inclusive of breaks. After providing informed consent participants completed the assessment battery. Except for the informant-rated measures (which were always completed at a later time point), these assessments were counterbalanced.

#### Social frailty

##### The Social Frailty Scale

(Pek et al., 2020.) Although most measures of social frailty do not have a clear conceptual basis guiding their development, a notable exception is the Social Frailty Scale, with this eight-item scale constructed to index social frailty as defined in Bunt et al.’s (2017) model. All questions require yes/no answers (e.g., “Do you sometimes visit your friends?”), with three items reverse-coded. Scores range from 0 to 8, with higher scores indicative of greater social frailty. Although Pek et al. (2020) also suggest social frailty categories, in line with theoretical frameworks that regard social frailty as lying along a continuum, all analyses in this study treat social frailty as a continuous variable. Scores on this measure have been linked to mood, nutrition, physical performance, and physical activity, independently of physical frailty (Pek et al., 2020).

#### Social cognition

##### Social perception

Failures of social perception typically manifest as problems recognizing and responding to basic social and emotional cues such as changes in eye contact, body posture, and movement. Because in broader social cognitive aging literature social perception has been most frequently assessed by asking participants to identify facial expressions, in the present study stimuli from The Amsterdam Dynamic Facial Expression Set—Bath Intensity Variations (ADFES-BIV; Wingenbach et al., 2016) were used to index this capacity. The ADFES-BIV is a standardized and validated set of dynamic facial expressions. A 40-trial version was used which included four actors (two male, two female) expressing the six basic emotions (anger, sadness, happiness, disgust, surprise, and fear), three self-conscious emotions (contempt, pride, and embarrassment), and a neutral facial expression at moderate intensity. Trials were presented in a randomized order, with each video presented for one second. For each trial, participants are asked to select the emotion that best describes the expression shown from 10 possible answers. The total score out of 40 was converted to a percentage to reflect overall accuracy on the task.

##### Theory of mind

ToM refers to the capacity to understand others’ mental states, and to appreciate that these may differ from our own. In the present study The Reading the Mind in the Eyes Test (RMET; Baron-Cohen et al., 2001) was used because it is a sensitive measure of relatively basic mental state decoding. The RMET imposes minimal demands on “higher level” cognitive control operations, such as working memory and abstract reasoning, that are commonly required in other measures of ToM, and which make age-related ToM difficulties more difficult to interpret (Henry et al., in press). Participants are required to view a series of photographs and infer how the protagonist is feeling by selecting one of four possible affective or mental states (e.g., serious, ashamed, alarmed, bewildered). The RMET includes 36 trials in total, each scored as correct or incorrect, and total scores were converted to percentage to reflect overall accuracy.

##### Affective empathy

The Questionnaire of Cognitive and Affective Empathy (QCAE; Reniers et al., 2011) is a 31-item self-report questionnaire that assesses empathy across cognitive and affective domains. For this study, only the 12 items that indexed affective empathy were included (e.g., “I often get emotionally involved with my friend’s problems”). Although affective empathy has been defined in several different ways (see Hall & Schwartz, 2019), most agree that it involves an emotional response toward another individual. In the present study, the QCAE was chosen because it was developed to assess the ability to be sensitive to, and vicariously experience, the emotional states of others. It has also been shown to have good psychometric properties and has been validated for use in older adult cohorts (Reniers et al., 2011). Scores are recorded on a 4-point Likert scale, with total scores ranging from 12 to 48 (higher scores indicate greater affective empathy).

##### Social behavior

Because people are often quite poor at judging the appropriateness of their own social behavior (presumably because they typically would not engage in socially inappropriate behavior if they knew it was inappropriate), some of the strongest evidence on age effects has been provided by informant-rated measures (see Henry et al., in press). In the present study, the informant-rated Socioemotional Dysfunction Scale (SDS; Barsuglia et al., 2014) was therefore used to index social behavior. Participants were instructed to ask someone who knows them well (e.g., a close friend or family member) to complete this measure on their behalf and return it to the research team (see Supplementary Table 2 for a breakdown of informant demographics and participant relationship). The SDS includes 40 statements that are designed to measure a range of behaviors that include extraversion, warmth, social influence, insight, openness, appropriateness, and maladjustment. Informants were required to rate each statement on a 5-point scale, ranging from 1 (very inaccurate) to 5 (very accurate). The SDS provides a global score of social competencies, with scores ranging from 40 to 160, and higher scores indicative of greater social dysfunction.

#### Positive psychological well-being predictors and indicators

##### Resilience

The short-form Resilience Scale (Wagnild, 2009) is a 14-item measure that assesses physical, cognitive, emotional, social, and spiritual resilience. Participants responded on a 7-point Likert-type scale ranging from 1 (*strongly disagree*) to *7* (*strongly agree*), with a sum score range of 14–98 (where higher scores indicated higher resilience). It has been extensively validated in diverse adult populations and shown to have good psychometric properties (Mirosevic et al., 2019).

##### Life satisfaction

The Satisfaction with Life Scale (Diener et al., 1985) is a five-item measure of life satisfaction that assesses global cognitive judgements of satisfaction with one’s life. Participants responded on a 7-point Likert-type scale from 1 (*strongly disagree*) to 7 (*strongly agree*), with a sum score range of 5–35 (higher scores indicate higher satisfaction). This measure has been extensively validated in diverse adult populations and has good psychometric properties.

#### Negative psychological well-being indicators

##### Demoralization

 The Demoralization Scale (Kissane et al., 2004) is a 24-item measure that assesses the extent to which individuals have experienced loss of meaning and purpose, dysphoria, disheartenment, helplessness, and sense of failure in the last two weeks. Participants respond on a 5-point Likert scale from 0 (*never*) to 5 (*all the time*), with a sum score range of 0–96 (where higher scores indicate more severe demoralization). Although depression is closely related to demoralization, a recent study of 1,527 cancer patients using exploratory graph analysis revealed that, apart from suicide ideation and fear of failure, depressive symptoms cluster in a distinct and stable community clearly separated from demoralization, with loss of hope and meaning, poor coping, and feelings of entrapment sitting centrally as core symptoms that generate the state of demoralization (Bobevski et al., 2022).

##### Social anxiety

The Self-Report Liebowitz Social Anxiety Scale (LSAS; Liebowitz, 1987) is a 24-item measure that separately assesses fear and avoidance in social interaction (e.g., meeting strangers) and performance (e.g., making a phone call in public) scenarios. Participants respond on two separate 4-point Likert-type scales to assess the extent of fear and avoidance in 24 different scenarios. Scoring for each item ranges from 0 (none) to 3 (severe), with a sum score range of 0–144 (where higher scores indicate more severe social anxiety). This measure has good psychometric properties (Heimberg et al., 1999).

##### Apathy

The Dimensional Apathy Scale (Radakovic & Abrahams, 2014) is a 24-item measure of apathy that assesses three aspects of goal-directed behavior (auto-activation, cognitive, and emotional) over the last month. Participants respond on a 4-point Likert-type scale from 0 (*hardly ever*) to 3 (*almost always*), with a sum score range of 0–72 (where higher scores indicate more severe apathy).

#### Control variables

To test the specificity of any relationships between social frailty with social cognition and the psychological well-being indicators or predictors, three control variables were included. The relevant subscales of The Tilburg Frailty Indicator (TFI; Gobbens et al., 2010) were used to measure physical frailty and cognitive frailty. For both the eight-item physical frailty subscale and the four-item cognitive frailty subscale, one point was assigned to each item, and items were summed to yield a total score between zero and eight, and zero and four, respectively (higher scores indicate higher levels of each respective type of frailty). Both these subscales have good psychometric properties and total TFI scores are associated with disability and quality of life (Gobbens & Uchmanowicz, 2021). The seven-item depression subscale of The Hospital Anxiety and Depression Scale (Zigmond & Snaith, 1983) was used to index depression. Scoring for each item ranges from 0 to 3, with a sum score range of 0–21 (higher scores indicate higher levels of depression).

### Analyses

The *Hmisc*, *QuantPsyc, rstatix*, *ppcor*, and *tidyverse* packages were used within RStudio (R version 4.1.0). To examine the relationship between social cognition and social frailty, two regression analyses were performed: (a) a standard multiple regression with the four social cognitive domains as the predictor variables and social frailty as the outcome variable, and (b) a hierarchical multiple regression to examine whether social cognition continued to predict social frailty after controlling for physical frailty, cognitive frailty, and depression at Step 1.

To examine the relationships between social frailty and the five well-being indices a series of regression analyses were performed whereby each well-being indicator was inputted separately as the outcome variable. First, five standard linear regressions were conducted to examine if social frailty significantly predicted well-being, and second, five hierarchical multiple regressions were run to examine if these relationships held after controlling for physical frailty, cognitive frailty, and depression at Step 1.

Missing data occurred in three measures (10 participants’ data from the informant rated SDS, 53 values across four participants in the LSAS, and one value from one participant in the cognitive frailty index of the TFI). In these instances, the mean response was imputed. In cases where multiple informant responses were provided for a single participant, the following sequential steps were taken to choose one informant: (a) fewer missing data; (b) partner over other relationships; (c) most regular physical contact; and (d) relationship length.

## Results

### Social Cognition as a Predictor of Social Frailty

The overall standard multiple regression model was significant (*F*(4,85) = 4.35, *p* = .003), with 16.98% of the variance in social frailty explained by the four social cognitive domains (adjusted *R*2 = .13). However, as given in Table 1, only affective empathy and social behavior emerged as significant predictors, with a greater propensity for undesirable social behavior and lower affective empathy associated with greater social frailty. Together, the three control measures explained 11.11% of the variance in social frailty at Step 1 (*F*(3, 86) = 3.98, *p* = .011, adjusted *R*^2^ = .080); however as shown in Table 2, only depression emerged as a significant predictor. At Step 2, the four social cognitive measures explained an additional 12.70% of variance (*R*^2^ = .24, adjusted *R*^*2*^ = .17). The overall model was significant (*F*(7, 82) = 3.66, *p* = .002). Depression remained significantly and positively related to social frailty at Step 2. Of the specific social cognitive domains, only poor social behavior made a significant unique contribution to the model, with poor social behavior and social frailty positively related.

**Table 1.** Descriptive Statistics, Correlations, and Regression Effect Sizes for Social Cognition Predicting Social Frailty

Statistic	Social frailty (outcome)	Social cognitive dimensions (Predictors)			
		Social perception (%)	Theory of mind (%)	Affective empathy	Inappropriate social behavior
*M* (*SD*)	1.63 (1.59)	58.94 (12.87)	73.89 (10.61)	30.36 (4.37)	66.80 (20.92)
Social frailty correlation (*r*)	—	.17	.06	−.23*	.31**
β (95% CI)	—	0.17 (−0.03, 0.37)	0.07 (−0.13, 0.27)	−0.21* (−0.41, −0.01)	0.29** (0.09, 0.49)
*sr* ^2^	—	.027	.004	.043	.078

*Notes*: CI = confidence interval, *sr*^2^ = squared semipartial correlation coefficient. The overall model was significant (*F*(4,85) = 4.35, *p* = .003), with 16.98% of the variance in social frailty explained by the social cognition domains (13.07% adjusted *R* squared). However, only affective empathy and social behavior significantly predict the relationship.

*N* = 90. *p < .05, **p < .01.

**Table 2.** Hierarchical Multiple Regression Results for Social Cognition Predicting Social Frailty

	β	95% CI for β	*sr* ^2^
Step 1			
Physical frailty	−0.21	−0.43, 0.01	.039
Cognitive frailty	−0.10	−0.33, 0.13	.008
Depression	0.36**	0.13, 0.60	.096
Step 2			
Physical frailty	−0.14	−0.35, 0.08	.014
Cognitive frailty	−0.05	−0.28, 0.18	.002
Depression	0.30*	0.07, 0.53	.062
Social perception	0.15	−0.05, 0.35	.021
Theory of mind	0.07	−0.12, 0.27	.005
Affective empathy	−0.20	−0.41, 0.00	.035
Inappropriate social behavior	0.24*	0.04, 0.44	.053

*Notes*: CI = confidence interval, *sr*^2^ = squared semipartial correlation coefficient.

*N* = 90. *p < .05, **p < .01.

### Social Frailty as a Predictor of Psychological Well-Being

As shown in Table 3, social frailty was consistently related to the five predictors and indicators of well-being. Specifically, social frailty significantly predicted greater apathy (*F*(1, 88) = 6.42, *p* = .013, *R*^2^ = .07, adjusted *R*^*2*^ = .06), demoralization (*F*(1, 88) = 18.46, *p* < .001, *R*^*2*^ = .17, adjusted *R*^*2*^ = .16), social anxiety (*F*(1, 88) = 6.50, *p* = .013; *R*^*2*^ = .07, adjusted *R*^*2*^ = .06), lower resilience (*F*(1, 88) = 11.14, *p* = .001, *R*^*2*^ = .11, adjusted *R*^*2*^ = .10), and poorer life satisfaction (*F*(1, 88) = 29.15, *p* < .001, *R*^*2*^ = .25, adjusted *R*^*2*^ = .24). However, as shown in Table 4, these relationships only held for demoralization, resilience, and satisfaction with life, after controlling for physical frailty, cognitive frailty, and depression in Step 1. While physical frailty failed to significantly predict well-being across all five indices, both cognitive frailty and depression often emerged as significant unique contributors (at both Step 1 and Step 2).

**Table 3.** Descriptive Statistics, Correlations, and Regression Effect Sizes for Social Frailty Predicting Well-Being Indicators

Outcomes	*M*	Social frailty predictor statistics		
	(*SD*)	*r*	β (95% CI)	*sr* ^2^
Apathy	23.37 (6.81)	.26**	0.26* (0.06, 0.47)	.068
Demoralization	20.79 (10.15)	.42***	0.42*** (0.22, 0.61)	.173
Social anxiety	30.06 (18.25)	.26*	0.26* (0.06, 0.47)	.069
Resilience	82.69 (8.69)	−.34**	−0.34** (−0.54, −0.14)	.112
Satisfaction with life	25.38 (6.13)	−.50***	−0.50*** (−0.68, −0.32)	.249

*Notes*: CI = confidence interval, *sr*^2^ = squared semipartial correlation coefficient. A separate analysis was completed for each outcome variable.

*N* = 90. *p < .05, **p < .01, ***p < .001.

**Table 4.** Hierarchical Multiple Regression Results for Social Frailty Predicting Each Well-Being Indicator

	Apathy			Demoralization			Social anxiety			Resilience			Satisfaction with life		
	β	95% CI for β	*sr* ^2^	β	95% CI for β	*sr* ^2^	β	95% CI for β	*sr* ^2^	β	95% CI for β	*sr* ^2^	β	95% CI for β	*sr* ^2^
Step 1	*F*(3, 86) = 4.44, *p* = .006 *R*^2^ = .13, adjusted *R*^2^ = .10			*F*(3, 86) = 21.45, *p <* .001 *R*^2^ = .43, adjusted *R*^2^ = .41			*F*(3, 86) = 4.49, *p* = .003 *R*^2^ = .15, adjusted *R*^2^ = .12			*F*(3, 86) = 8.12, *p* < .001 *R*^2^ = .22, adjusted *R*^2^ = .19			*F*(3, 86) = 8.52, *p* < .001 *R*^2^ = .23, adjusted *R*^2^ = .20		
Physical frailty	−0.19	−0.40, 0.03	.030	−0.10	−0.27, 0.08	.008	−0.13	−0.35, 0.08	.015	0.09	−0.11, 0.30	.008	0.14	−0.07, 0.34	.016
Cognitive frailty	0.10	−0.13, 0.33	.008	0.33***	0.14, 0.52	.082	0.09	−0.14, 0.32	.006	−0.24*	−0.46, −0.03	.045	−0.33**	−0.55, −0.11	.082
Depression	0.34**	0.10, 0.57	.083	0.47***	0.28, 0.66	.159	0.37**	0.13, 0.60	.097	−0.34**	−0.56, −0.44	.082	−0.27*	−0.49, −0.05	.053
Step 2	*F*(4, 85) = 4.13, *p* = .004 *R*^2^ = .16, adjusted *R*^2^ = .12			*F*(4, 85) = 23.45, *p* < .001 *R*^2^ = .52, adjusted *R*^2^ = .50			*F*(4, 85) = 4.57, *p* = .002 *R*^2^ = .18, adjusted *R*^2^ = .14			*F*(4, 85) = 8.58, *p* < .001 *R*^2^ = .29, adjusted *R*^2^ = .25			*F*(4, 85) = 15.76, *p* < .001 *R*^2^ = .43, adjusted *R*^2^ = .40		
Physical frailty	−0.15	−0.37, 0.07	.018	−0.03	−0.19, 0.14	.001	−0.10	−0.31, 0.12	.007	0.03	−0.17, 0.24	.001	0.04	−0.15, 0.22	.001
Cognitive frailty	0.12	−0.11, 0.35	.011	0.36***	0.19, 0.53	.099	0.11	−0.12, 0.34	.009	0.27*	−0.48, −0.06	.056	−0.38***	−0.57, −0.19	.107
Depression	0.27*	0.03, 0.51	.048	0.35***	0.16, 0.53	.080	0.30*	0.06, 0.54	.060	0.24*	−0.46, −0.01	.036	−0.10	−0.30, 0.10	.007
Social frailty	0.18	−0.03, 0.39	.028	0.33***	0.17, 0.49	.097	0.18	−0.03, 0.39	.029	0.27**	−0.47, −0.08	.067	−0.47***	−0.64, −0.30	.196

*Notes:* CI = confidence interval, *sr*^2^ = squared semipartial correlation coefficient.

*N* = 90. *p < .05, **p < .01, ***p < .001.

## Discussion

These data provide novel evidence about social cognitive correlates of social frailty, as well as how inadequate social needs fulfillment is related to psychological characteristics known to be important for health and well-being in older age. First, lower self-rated affective empathy and poorer social behavior as rated by participants’ own peers were both associated with increased social frailty, with this latter relationship remaining significant even after controlling for physical and cognitive components of frailty as well as depression. Second, social frailty predicted all five indices of psychological well-being, and for resilience, demoralization, and satisfaction with life, these relationships remained significant even after controlling for covariates.

### Social Cognitive Correlates of Social Frailty

The finding of relationships between affective empathy and social behavior with social frailty suggests that, even in the context of normal adult aging, social cognitive difficulties may be associated with meaningful losses in social capital. However, perhaps surprisingly, neither social perception or ToM were linked to social frailty. These two sets of findings might seem contradictory given that social cognitive processes work together to allow people to understand and manage others, as is reflected in the development of many complex hierarchical conceptual models (Schurz et al., 2021). However, as noted previously, specific social cognitive skills do not appear to be affected equivalently by aging. For instance, even when other social cognitive abilities are intact, undesirable social behaviors may present independently. Declines in broader cognitive resources, such as executive control, increase older adults’ risk of engaging in inappropriate social behaviors (Henry et al., 2009), and these may then influence the responses of others and the individual’s capacity to manage social relationships. It is for this reason that social behavior is often regarded as part of, and not simply the endpoint of, social cognitive function (Henry et al., 2016).

Nevertheless, a key point to acknowledge is the different methods that were used to index the four social cognitive domains. Only the measure of social behavior was informant-rated and given there is research showing that others may sometimes know us better than we know ourselves (Vazire & Carlson, 2011), this might explain why only social behavior emerged as a unique predictor of social frailty. Moreover, for the two social cognitive domains that showed no association with social frailty (social perception and ToM), participants were presented with stimuli depicting or describing unknown individuals, which they were then asked to evaluate or respond to in some manner. While this is the predominant method used in studies of social cognitive aging, these types of stimuli may or may not tap into the social cognitive skills older adults use in everyday life (see Henry et al., in press). Such skills may be better captured by the methods used to index affective empathy (which asked about participants’ real-life emotional responses) and social behavior (which enquired about participants’ ability to engage with real-life social partners in familiar contexts). Having now established that conventional lab-based measures of social perception and ToM are not associated with social frailty in older age, the next important step is to test these relationships using more ecologically valid approaches that tap into how these abilities are engaged in actual, daily life.

These points noted, the current study provides important initial evidence that normal variation in social behavior may be valuable for understanding social frailty in older age. Although undesirable social behaviors such as insensitive or inappropriate remarks are frequently not meant to be off-putting—as they are often a consequence of poor social skills—they are nevertheless widely interpreted as unfriendly (Riggio, 1986). Moreover, people find the company of others who regularly engage in socially undesirable behaviors to be aversive and tend to avoid such individuals. All prominent models of socioemotional aging also emphasize how, with increasing age, people become more selective in how they invest their social resources, with an emphasis on positive emotional returns (Fung et al., 2020), with this pattern even extending to digital social networks (Chiarelli & Batistoni, 2021). According to socioemotional selectivity theory, altered time perspective explains why older people are more motivated to pursue positive and avoid negative social interactions (Carstensen, 2021), while the selective engagement hypothesis suggests that decline in the availability of resources needed to support social behavior contributes to increased social selectivity (Hess, 2014). The social cognitive resource framework (Henry et al., in press) additionally highlights the central role of prior experience in determining social resource allocation. All these models (and many others) converge in predicting that less desirable social behaviors in late adulthood may have more important social “costs” than in younger adulthood—and the current study suggests that one of the ways in which these costs may present is via increased social frailty.

### Psychological Consequences of Social Frailty

The results from this study also provide novel insights into how social frailty relates to psychological characteristics known to be important for health and well-being in older age. As anticipated, social frailty was a significant predictor of resilience, life satisfaction, demoralization, apathy, and social anxiety, and for the former three psychological characteristics, these effects remained significant even after controlling for the broader effects of physical frailty, cognitive frailty, and depression. (It should be noted that a limitation in interpreting these findings was that the measure of cognitive frailty had low internal consistency.) The negative relationship identified with life satisfaction cross-validates prior literature (Ko & Jung, 2021), and directly aligns with predictions from Bunt et al.’s (2017) model, in which a central tenet is that adequate fulfillment of basic social needs is critical for subjective well-being, including life satisfaction. However, this study is the first to show that social frailty is also associated with both psychological resilience and demoralization.

Models of socioemotional aging such as The Strength and Vulnerability Integration (Charles, 2010) framework highlight how psychological resilience may become increasingly important with age. Although older adults may have adequate coping skills for familiar stressors such as interpersonal conflict or work stress, the novelty of many stressors more likely to be encountered in older age (such as bereavement, or physical illness), may reduce the effectiveness of these coping skills, making psychological resilience to adversity more critical. Consistent with such models, psychological resilience is more consistently and robustly associated with health transitions and trajectories than many other commonly used resource indicators (Taylor & Carr, 2021). Resilience has also emerged as an important target for successful aging in broader literature, not only because it is linked with mechanistic processes and outcomes that contribute to healthy aging, but because it is measurable and modifiable (Kim et al., 2021).

The finding that higher levels of social frailty were also predictive of greater demoralization also provides important novel evidence about the psychological mechanisms by which social frailty might contribute to so many negative health and well-being outcomes. Unlike the loss of momentary pleasure (anhedonia) that is a core feature of depression, demoralization describes a syndrome of existential distress and despair. Yet despite its profound impact on well-being, demoralization has been the focus of only very limited empirical attention in broader gerontological literature, with most research instead focused on clinical populations.

Only one study to date speaks to whether vulnerability to demoralization might change with age. In this representative general population cohort of more than 2,000 participants aged between 18 and 94 years, Quintero Garzon et al. (2021) identified no association between age and demoralization. However, a substantial proportion of the participants sampled (13.5%) scored ≥30, indicative of a clinically relevant moderate level of demoralization (Kissane et al., 2004). Interestingly, a very similar proportion of the older adults in the current study also scored above this cut-off (14.4%), while a recent systematic review of demoralization in clinical populations also revealed that being single or socially isolated were important risk factors (Robinson et al., 2015). Taken together, the current study coupled with prior literature therefore suggests not only that demoralization is a relatively common syndrome in adult cohorts even in the absence of serious clinical illness, but that inadequate social needs fulfillment may be an important risk factor for this debilitating syndrome.

With respect to how social frailty might be linked to poorer life satisfaction, reduced resilience, and increased demoralization, functional and structural accounts have been proposed to explain how social resources might be protective in older age. Whereas the stress-buffering hypothesis (Cohen, 2004) focuses on functional aspects of social relationships, and how the subjective experience of social support mitigates the psychological and physiological impact of stress, direct-effect frameworks emphasize more structural aspects of social support such as network size. Here, the view is that social support has a direct positive effect on health and well-being irrespective of stress level because it increases the likelihood of social engagement and exposure to positive health information. Indeed, a central tenet of Cacioppo et al.’s (2011) Social Control Hypothesis is that social interactions increase health behavior, and consistent with this causal pathway frailty has been directly linked to poorer health behavior (Geboers et al., 2016; Gil-Salcedo et al., 2020). Future research is now needed to establish whether specific types of social resources differentially impact these two possible pathways. Indeed, while prior research has consistently identified strong relations between social frailty with important health and well-being outcomes, this is also true for more specific types of social vulnerability status (such as social isolation and loneliness), and it remains unclear whether the lack of very specific social resources on health and well-being is independent, or additive.

## Conclusion

To conclude, these data provide the first evidence that some but not all aspects of social cognitive function are predictive of social frailty and affords novel insights into how social frailty is related to key psychological characteristics known to be important for health and well-being in older age.

## Supplementary Material

gbac157_suppl_Supplementary_Data
