# Supplementary material for: Social Frailty in Late Adulthood: Social Cognitive and Psychological Well-Being Correlates
Source: J Gerontol B Psychol Sci Soc Sci. 2022 Oct 3;78(1):87–96. doi: 10.1093/geronb/gbac157 (PMC9890915; doi:10.1093/geronb/gbac157)
Supplement: gbac157_suppl_Supplementary_Data [file gbac157_suppl_supplementary_data.docx]

**Supplementary Table 1.** Participant Demographic Information

| **Characteristic** | **Statistic** |
| --- | --- |
|  | ***M (SD)*** |
| ACE score | 28.16 (1.77) |
| Age (years) | 73.78 (5.92) |
| Education (years) | 14.84 (4.11) |
|  | ***n* (%)** |
| **Gender** |  |
| Male | 45 (50.00) |
| Female | 45 (50.00) |
| **Ethnicity** - Caucasian | 90 (100.00) |
| **Relationship Status** |  |
| Single | 9 (10.00) |
| De Facto | 4 (4.44) |
| Married | 49 (54.44) |
| Widowed | 7 (7.78) |
| Divorced/Separated | 20 (22.22) |
| Other | 1 (1.11) |
| **Living Arrangements** |  |
| Alone | 31 (34.44) |
| Spouse | 53 (58.89) |
| Other Relatives | 2 (2.22) |
| **Characteristic** | **Statistic** |
|  | ***n* (%)** |
| **Living Arrangements (continued)** |  |
| In Community Setting | 1 (1.11) |
| Housemate | 2 (2.22) |
| Other | 1 (1.11) |
| **Employment Status** |  |
| Retired | 76 (84.44) |
| Working Part-Time | 10 (11.11) |
| Unemployed | 1 (1.11) |
| Other | 3 (3.33) |
| **History of Mental Health** |  |
| Yes | 15 (16.67) |
| No | 75 (83.33) |
| **Self-Health Rating** |  |
| Poor | 2 (2.22) |
| Fair | 13 (14.44) |
| Good | 38 (42.22) |
| Very Good | 27 (30.00) |
| Excellent | 10 (1.11) |

*N* = 90. ACE = Addenbrooke's Cognitive Examination.

**Supplementary Table 2.** Informant Demographic and Relationship Information

| **Characteristic** | **Statistic** |
| --- | --- |
|  | ***M (SD)*** |
| Age (years) | 66.32 (13.89) |
| Education (years) | 15.46 (3.33) |
| Relationship Length (years) | 39.10 (18.00)^a^ |
| Self-Reported Knowledge^b^ | 6.63 (0.64)^a^ |
| Self-Reported Closeness^b^ | 6.53 (0.77)^a^ |
|  | ***n* (%)** |
| **Gender** |  |
| Male | 32 (40.00) |
| Female | 48 (60.00) |
| **Relationship Type**^a^ |  |
| Friend | 22 (27.85) |
| Relative | 18 (22.78) |
| Partner | 39 (49.37) |
| **Live with Participant**^a^ |  |
| Yes | 41 (48.10) |
| No | 38 (51.90) |
| **If no, Frequency of Physical Contact** | |
| Most Days | 6 (15.79) |
| Once a Week | 13 (34.21) |
| **Characteristic** | **Statistic** |
|  | ***n* (%)** |
| **If no, Frequency of Physical Contact (continued)** | |
| Once a Fortnight | 7 (18.42) |
| Once a Month | 2 (5.26) |
| Every Few Months | 6 (15.79) |
| Once a Year (or less) | 4 (10.53) |

*N* = 80. ^a^N = 79. ^b^scored on a 7-point scale from not at all to extremely well/close.

**Supplementary Table 3.** Control Measures Descriptive Statistics and Bivariate Correlations with Social Frailty

| **Control Measure** | ***M (SD)*** | ***r*** |
| --- | --- | --- |
| Physical Frailty | 1.51  (1.49) | -.11 |
| Cognitive Frailty | 0.94  (0.93) | .00 |
| Depression | 3.77  (2.35) | .24* |

*N* = 90. **p* < .05

An important limitation to interpreting these correlations is the self-report nature of the control measure. Although in a recent review of 42 frailty instruments, the Tilburg Frailty Index was identified as one of two measures that could be generally recommended as a screening tool for frailty (Huang & Lam, 2021), as with all self-report methods, it is not able to assess cognition in a fine-grained fashion. Future research focused on better understanding the nature of the relationship between social cognition and social frailty should therefore include an objective test battery that taps into a range of cognitive abilities. Future research should also endeavour to assess physical frailty using objective assessments.

**References**

Huang, E. Y., & Lam, S. C. (2021). Review of frailty measurement of older people: Evaluation of the conceptualization, included domains, psychometric properties, and applicability. *Aging Med (Milton), 4*(4), 272-291. doi:10.1002/agm2.12177

**Supplementary Table 4**. Reliability Estimates for all Self- and Informant-Report Measures.

| **Measure** |  | **Omega**  **(**$\boldsymbol{\omega}$**)** |
| --- | --- | --- |
|  |  |  |
|  |  |  |
| Social Frailty Scale |  | .78 |
| QCAE Affective Empathy Scale |  | .76 |
| Social Dysfunction Scale |  | .96 |
| Dimensional Apathy Scale |  | .81 |
| Demoralisation Scale |  | .95 |
| Short-form Resilience Scale |  | .92 |
| Satisfaction with Life Scale |  | .94 |
| Tilburg Physical Frailty Scale |  | .66 |
| Tilburg Cognitive Frailty Scale |  | .40 |
| Hospital Anxiety Depression Scale |  | .78 |
|  |  |  |

In line with the growing literature describing Macdonald’s omega as a more appropriate index of reliability than Cronbach’s alpha (e.g., McNeish, 2018; Watkins, 2017), omega ($\omega$) was used to estimate the reliability of each self-report scale in the present study. The estimate was calculated using the *psych*(Revelle, 2022) package in R, which uses a more sophisticated variance decomposition (McNeish, 2018).  Watkins (2017) notes that omega coefficients should meet the same standards as alpha, with .50 often reported as a minimum standard (Reise, 2012; Taber, 2015), but .70 generally preferred (Nunnally & Bernstein, 1994)

**References**

McNeish, D. (2018). Thanks coefficient alpha, we’ll take it from here. *Psychological Methods*, *23*(3), 412-433. <https://doi.org/10.1037/met0000144>

Nunnally, J. C., & Bernstein, I. H. (1994). *Psychometric theory*(3rd ed.). McGraw-Hill.

Reise, S. P. (2012). The rediscovery of bifactor measurement models. *Multivariate Behavioral Research,* *47*, 667–696. <https://doi.org/10.1080/00273171.2012.715555>

Revelle, W. (2022) *psych: Procedures for Personality and Psychological Research*, Northwestern University, Evanston, Illinois, USA,

[https://CRAN.R-project.org/package=psych](https://cran.r-project.org/package=psych) Version = 2.2.5.

Taber, K. S. (2018). The use of Cronbach’s alpha when developing and reporting research instruments in science education. *Research in science education, 48*(6), 1273-1296. <https://doi.org/10.1007/s11165-016-9602-2>

Watkins, M. W. (2017). The reliability of multidimensional neuropsychological measures: From alpha to omega. *The Clinical Neuropsychologist*, *31*(6-7), 1113 1126. https://doi.org/10.1080/13854046.2017.1317364
